# Thumb and finger movement is reduced after stroke: An observational study

**DOI:** 10.1371/journal.pone.0217969

**Published:** 2019-06-12

**Authors:** Helleana Eschmann, Martin E. Héroux, James H. Cheetham, Stephanie Potts, Joanna Diong

**Affiliations:** 1 Faculty of Health Sciences, University of Sydney, Lidcombe, NSW, Australia; 2 Neuroscience Research Australia (NeuRA), Randwick, NSW, Australia; 3 University of New South Wales, Randwick, NSW, Australia; 4 Physiotherapy Department, Prince of Wales Hospital, Randwick, NSW, Australia; 5 School of Medical Sciences, Faculty of Medicine and Health, University of Sydney, Sydney, NSW, Australia; University of Massachusetts Amherst, UNITED STATES

## Abstract

Hand motor impairment is common after stroke but there are few comprehensive data on amount of hand movement. This study aimed to compare the amount of thumb and finger movement over an extended period of time in people with stroke and able-bodied people. Fifteen stroke subjects and 15 able-bodied control subjects participated. Stroke subjects had impaired hand function. Movement of the thumb and index finger was recorded using stretch sensors worn on the affected hand (stroke subjects) or the left or right hand (control subjects) for ∼4 hours during the day. A digit movement was defined as a monotonic increase or decrease in consecutive sensor values. Instantaneous digit position was expressed as a percentage of maximal digit flexion. Mixed linear models were used to compare the following outcomes between groups: (1) average amplitude of digit movement, (2) digit cadence and average digit velocity, (3) percentage of digit idle time and longest idle time. Amplitude of digit movement was not different between groups. Cadence at the thumb (between-group mean difference, 95% CI, p value: -0.6 movements/sec, -1.0 to -0.2 movements/sec, p = 0.003) and finger (-0.5 movements/sec, -0.7 to -0.3 movements/sec, p<0.001) was lower in stroke than control subjects. Digit velocity was not different between groups. Thumb idle time was not different between groups, but finger idle time was greater in stroke than control subjects (percentage of idle time: 6%, 1 to 11%, p = 0.02; longest idle time: 375 sec, 29 to 721 sec, p = 0.04). Rehabilitation after stroke should encourage the performance of functional tasks that involve movements at faster cadences, and encourage more frequent movement of the digits with shorter periods of inactivity.

## Introduction

Motor impairment at the hand is common after stroke [1]. At 6 months after severe stroke, one third of people develop wrist and hand contracture (loss of passive joint range of motion) [2] and more than 50% of people with hand impairments do not regain function [3]. The loss of functional hand movement is disabling and can persist for many years [4]. The neurophysiological mechanisms underlying the recovery of hand function are complex, interdependent, and occur at different periods of time after onset of stroke; see reviews [5–7]. For example, hand impairments in chronic stroke are related in part to a decreased ability to control voluntary muscle activity [8, 9] and the abnormal recruitment of contralateral cortico-reticulospinal pathways [10]. Greater understanding on the mechanisms of recovery after stroke is needed to develop effective interventions to improve hand function after stroke.

Clinical rehabilitation after stroke aims to improve upper limb function through high-intensity, task-specific practice [11, 12]. These principles are implemented in clinical practice guidelines, which recommend that therapists encourage people with stroke to use their affected hand during rehabilitation to improve strength and functional recovery [13]. These recommendations are based on the view that functional recovery is driven by physical and behavioral adaptations in response to weakness, loss of dexterity, and other impairments after stroke. That is, both neurophysiological mechanisms and learned non-use of the affected limb [14, 15] contribute to persistent motor impairment. Findings from clinical trials support this view by showing that modified doses of constraint-induced movement therapy and robot-assisted training may improve arm function after stroke [11, 16]. However, these and other interventions do not improve *hand* function after stroke [17]. Why is this so? It may be that the dose of therapy is not sufficient to increase overall hand movement at the individual digits, especially if spastic dystonia or contracture are present. Thus, it is important to determine whether interventions to improve hand function deliver doses of therapy that are sufficiently high. To do so, we need methods to quantify the amount of thumb and finger movement in detail, over extended periods of time.

There are few data that comprehensively quantify the amount of thumb and finger movement after stroke. Movement of the affected arm measured with accelerometers indicates the affected arm is used up to 80% less than the unaffected arm [18, 19]. Movement of the affected arm can also be measured indirectly using activity mapping methods. Here, investigators observe stroke subjects at short, regular intervals over a day, and record tasks and activities that are performed. Overall, stroke subjects are mostly sedentary: they spend only 7% of the day standing or walking [20], and the average amount of time spent performing upper limb activities ranges from 0.9 to 7.9 minutes per therapy session [21]. Activity mapping is labor-intensive and is not well suited to monitor hand movement in detail in a clinical setting [19]. In addition, arm accelerometry and activity mapping methods do not show how much the hand is used. Indeed, a systematic review of methods to monitor physical activity in stroke did not find any study that assessed upper limb movement distal to the wrist [22]. Hand movements are important because they are used to dextrously manipulate objects to perform functional tasks.

To comprehensively capture hand movement, wearable sensors worn over the digits can be used [23, 24]. A number of small, proof-of-concept studies have been conducted to develop and validate these methods. In one study, stretch sensors over the metacarpophalangeal joints of the four fingers were worn by an able-bodied subject for 25 hours [25]. Investigators found that the fingers were relatively more extended when performing office work [mean (SD): 19° (10°)] and more flexed when performing self-care and household activities [28° (13°)]. In another study, an accelerometer at the wrist was combined with a magnetometer at the index finger to measure wrist and finger movement in 7 able-bodied subjects and 4 subjects with stroke [26]. Wrist movement was associated with finger movement in able-bodied subjects, and there was large variability in wrist and finger movement in subjects with stroke. However no between-group analyses were performed, movement at only one finger joint was examined, and only 4 subjects with stroke were tested. Two other studies used accelerometers worn at the index finger and wrist to measure hand movement over 8 hours in 10 able-bodied subjects [27, 28]. Sensor data correlated strongly with hand function and kinematic analysis of functional tasks. Lastly, pressure sensors were used to compare finger forces in 50 able-bodied subjects and 14 subjects with stroke [29]. Finger forces were associated with severity of stroke.

Overall, these studies show that novel yet simple wearable sensors can be used to measure digit movement and force in detail, both in people with stroke and able-bodied people. Indeed, wearable miniaturized sensors, robots and force sensors to measure kinematic and kinetic outcomes are welcomed in consensus-based core recommendations on standardized measurement of sensorimotor recovery in stroke trials [30]. However, there are insufficient data to make generalizable conclusions on hand movement after stroke. Furthermore, there are no published data comparing the amount of thumb and finger movement over long periods of time between people with stroke and able-bodied people. Without this information, it is not possible to understand in detail how hand movement after stroke differs from able-bodied people.

Therefore, this study aimed to compare the amount of thumb and finger movement over an extended period of time in people with stroke and able-bodied people. We used stretch sensors to measure thumb and index finger movement over 4 hours during the day. For people with stroke, this included therapy time if they were receiving inpatient or outpatient rehabilitation. We hypothesized that the thumb and finger move over a smaller range, move more slowly and less frequently in people with stroke compared to able-bodied people.

## Materials and methods

This was a cross-sectional observational study. The procedures conformed to the Helsinki Declaration and were approved by South Eastern Sydney Local Health District Human Research Ethics Committee (16/095). To enhance transparency of data analysis, all de-identified data and computer code used to analyse the data are available in the project folder on the Open Science Framework. Written consent was obtained from all subjects.

Fifteen subjects with stroke and 15 able-bodied control subjects were recruited. Subjects with stroke were recruited through the Department of Physiotherapy at the Prince of Wales Hospital. Subjects with stroke were included if they were at least 18 years old, had a medically-documented stroke of at least 2 weeks, able to speak and understand English, and had no history of fracture or orthopedic surgery at the hand, wrist or forearm. Stroke subjects were sampled broadly to obtain a representative sample of people with stroke, and could be receiving either inpatient or outpatient rehabilitation. Hand motor function was assessed using items 7 (Hand movements) and 8 (Advanced hand activities) of the Motor Assessment Scale [31]. Each item contains 6 tasks in increasing difficulty and subjects’ abilities to perform the tasks were scored from 0 (unable to perform task 1) to 6 (able to perform all tasks). The items were designed to assess hand function and include common tasks such as grasping a cup, fine pinch to grasp a small bean, using a pen, and using cutlery. The upper limb items of the Motor Assessment Scale have high test-retest and inter-tester reliability, and high construct validity [31, 32].

Control subjects were recruited through The University of Sydney, Neuroscience Research Australia (NeuRA) and the general community. Control subjects were included if they were at least 18 years old, able to speak and understand English, and had no history of fracture or orthopedic surgery at the hand, wrist or forearm.

### Protocol

**Fig 1:**
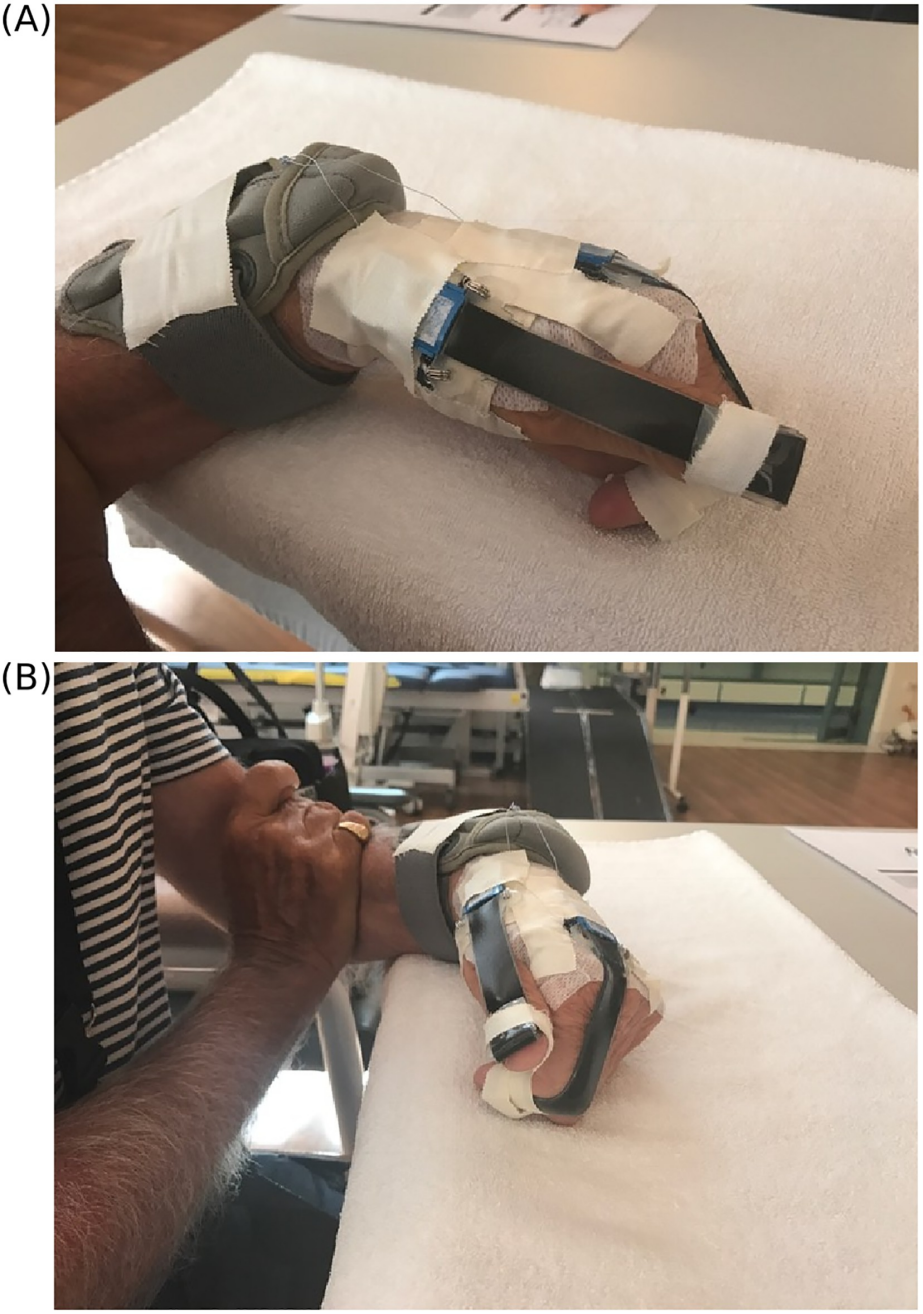
Stretch sensors worn on the left hand of a subject with stroke, showing the set-up in greater detail (A) at the thumb and (B) at the index finger. The thumb sensor was applied over the metacarpophalangeal and interphalangeal joints, and the index finger sensor was applied over the metacarpophalangeal and proximal interphalangeal joints. Each sensor was connected by cables to a data transmission system and battery in the pouch strapped below the wrist. Data were transmitted to an application on a smart phone carried on a lanyard or in the subject’s pocket (not shown).

A set of two commercial stretch sensors (StretchSense^™^, Auckland, New Zealand) was used to measure movement of the thumb at the metacarpophalangeal and interphalangeal joints, and movement of the index finger at the metacarpophalangeal and proximal interphalangeal joints. Measuring the combined motion of different joints provided an overall indication of movement of the whole digit. The stretch sensors were made from non-toxic, soft polymer, waterproof material. For each subject, sensors were applied over thumb and over the index finger with a small amount of pre-stretch (Fig 1). The ends of each sensor were attached over the dorsum of the digit using medical grade adhesive tape. Sensors were connected to a data transmission system and battery held in a small pouch strapped below the wrist. The data were transmitted wirelessly via Bluetooth to the StretchSense application on a smart phone. Raw data from the sensors were output in units of capacitance, sampled at 10 Hz, and stored as comma-separated-value (CSV) files on the smart phone. Subjects carried the smart phone on a lanyard or in their pocket to keep the phone within the range of Bluetooth connectivity.

The sensors were calibrated to determine range of motion of each digit. For each subject, the range through which the digits could move was measured in a calibration protocol where an investigator first passively held the digits in a neutral position (palm flat on table, thumb and index finger in extension) for 10 seconds, then passively flexed and held the index finger in full flexion for 10 seconds, and finally passively flexed and held the thumb in full flexion for 10 seconds. The calibration protocol was repeated 5 times and data were stored in a calibration file.

We originally intended to measure thumb and finger movement over 24 hours to obtain information on hand use over a full day. However, pilot testing showed that subjects with stroke found it uncomfortable to wear the sensors for longer than 4 hours. Thus, data were collected while the subject wore the sensors for 4 hours during the day. At the end of the 4 hour trial, a second calibration file was obtained.

### Data analysis

CSV files were exported and analysed offline using Python (v3.6). Maximal digit flexion range of motion was determined using maximal and minimal values from the 5 calibration protocols. During pilot testing, we noted that raw values from calibration protocols were not necessarily identical before and after the 4 hour trial. The sensors were applied to the skin under a small amount of stretch, so the ends of the sensors may have retracted slightly if there was perspiration, causing the calibration values to change. In addition, the investigator may not have maximally flexed the digits passively if subjects experienced pain or discomfort during the calibration protocols. Thus, maximal digit flexion range of motion was calculated using the average between values from the first and second calibration files.

Digit movements were determined using the raw sensor values. The change in values between consecutive samples was used to determine how far the digit moved in one direction before moving in the opposite direction. A single digit movement was defined as a monotonic increase or decrease in raw values of at least 1 capacitance unit between samples. Digit movements were expressed as a percentage of maximal digit flexion. Mixed linear models were used to compare the following outcomes between groups: (1) average amplitude of digit movement, (2) digit cadence (i.e. number of movements per second) and average digit velocity, and (3) percentage of digit idle time (i.e. time during which the digit is not moving) and longest idle time within the trial. Likelihood ratio tests were used to determine whether age was associated with outcomes. Sex differences between groups were compared using Fisher’s exact test. Mean between-group differences in outcomes and 95% CI are presented.

It is not known how digit movement is reflected in sensor raw values during task performance. That is, although a digit movement was defined as an increase or decrease in consecutive sensor values, it is not known how changes in sensor values correspond to dexterous digit movement in real-world, functional tasks. Thus, we explored how digit position changed during a functional task to provide reference values. An able-bodied subject wore the sensors and typed sentences on a computer keyboard at self-selected slow, medium and fast speeds, and the subject’s typing speeds were calculated from these data. For each subject in the study, the proportion of time that the digits spent moving at less than slow, between slow and medium, between medium and fast, and greater than fast speeds were then calculated for digit cadence and velocity outcomes. These exploratory data are described within groups using means (SD). In order to minimize Type I error, no between-group tests to make inferences were performed on these data.

## Results

Characteristics of subjects are shown (Table 1). Stroke subjects had impaired hand function, were older than control subjects, and the range of time since stroke was broad. There was no difference between groups in sex. On average, stroke subjects wore the sensors for median 3.3 hours (interquartile range 2.1 to 3.7 hours) and control subjects for 3.1 hours (2.3 to 3.7 hours). Inspection of the raw data showed sensor values were outside of the physiological range of raw values for the thumb sensor in some subjects (1 control, 2 stroke subjects). We excluded thumb sensor data for these subjects.

**Table 1:** Characteristics of control and stroke subjects. Data are shown as median (interquartile range) unless otherwise stated. P values indicate between-group differences. MAS: Motor Assessment Scale.

	Control (n = 15)	Stroke (n = 15)	p value
Age (years)	40 (17)*	76 (8)*	<0.001
	35 (25 to 61)	77 (72 to 82)	-
Male: female (n)	8 : 7	11 : 4	0.45
Right: left hand dominant (n)	13 : 2	15 : 0	-
Right: left side tested (n)	6 : 9	7 : 8	-
Ischemic: hemorrhagic stroke (n)	-	10 : 5	-
Right: left side affected side (n)	-	7 : 8	-
Time since stroke (months)	-	2.0 (1.1 to 3.6)	-
MAS item 7 (out of 6)	-	3.0 (0 to 5.5)	-
MAS item 8 (out of 6)	-	1.0 (0 to 4.0)	-

* Mean (SD)

**Fig 2:**
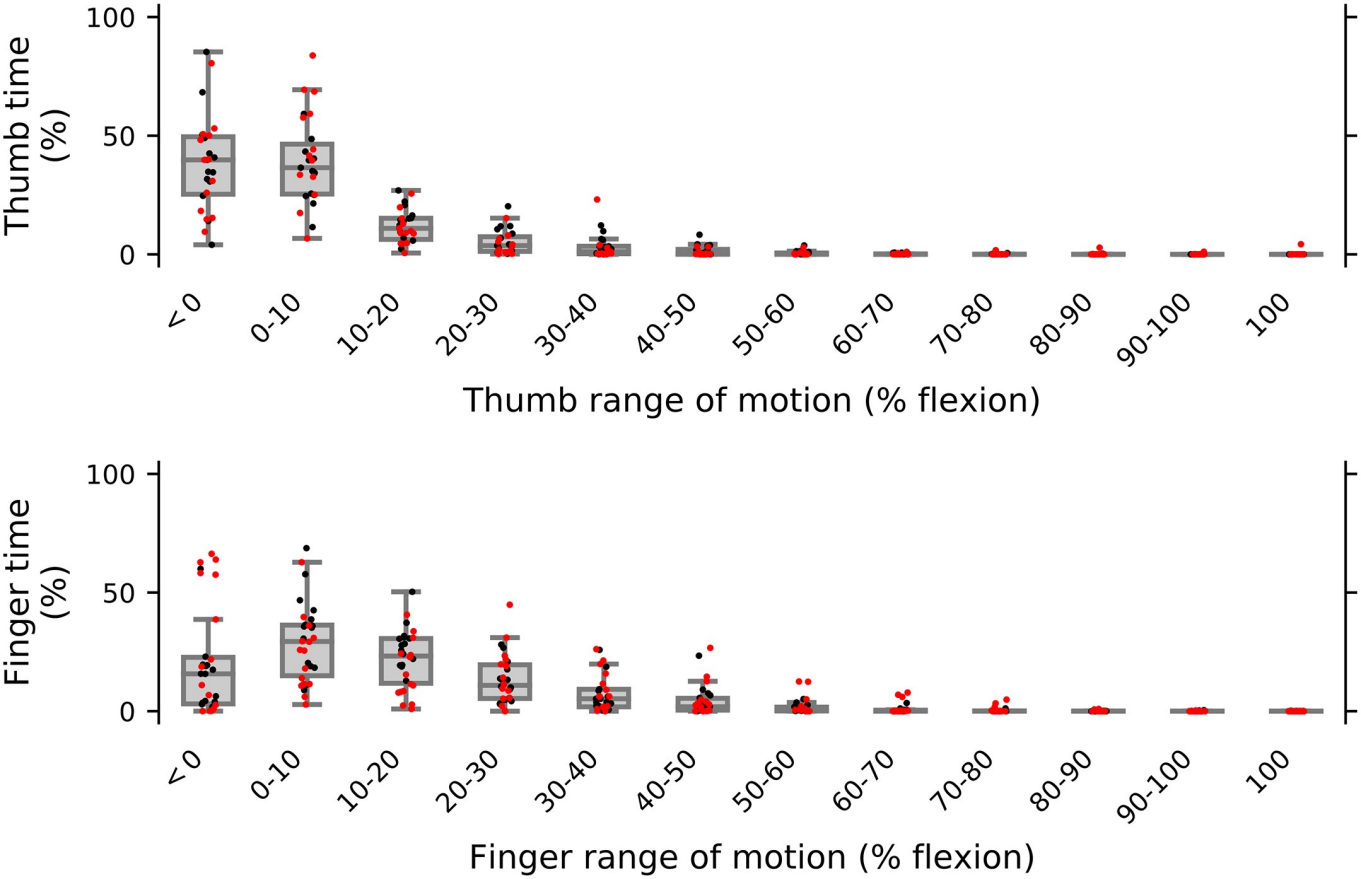
Percentages of time that the thumb (top panel) and index finger (bottom panel) spent at a given percentage of flexion range of motion. Median and interquartile ranges, and individual subject data for all stroke (red) and control (black) subjects are shown.

**Fig 3:**
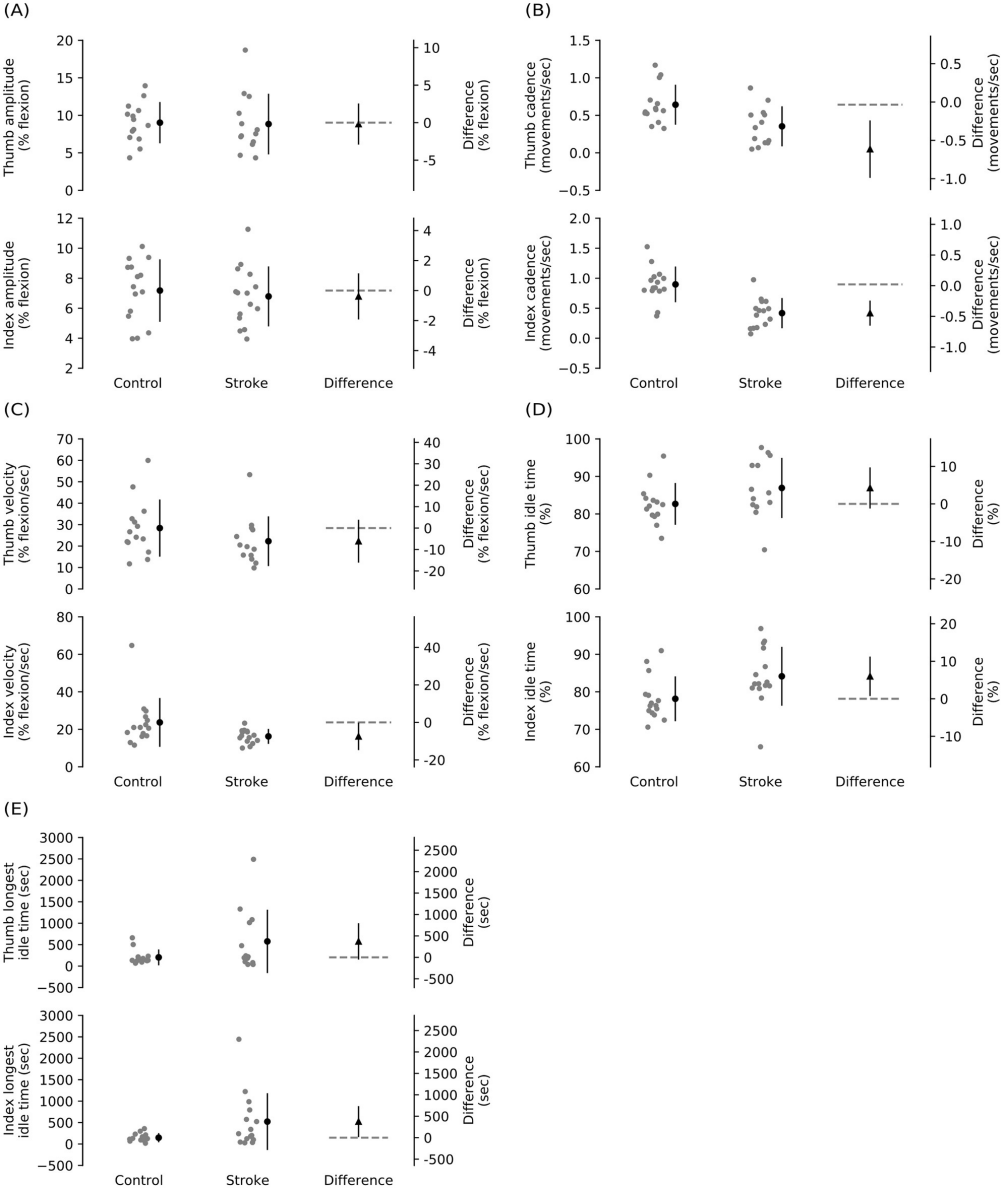
Plots of the main outcomes: (A) average amplitude of digit movement, (B) digit cadence, (C) average digit velocity, (D) percentage of digit idle time, (E) longest idle time. Individual subject data (gray circles) and means (SD) within groups (black circles and error bars), and between-group mean differences and 95% CI (triangle and error bars) with no difference of 0 shown (dashed line), at the thumb (top panel) and index finger (bottom panel).

The proportions of time spent by the digits at a given percentage of flexion range of motion are described (Fig 2). Both digits were relatively more extended over most of the trial. The percentages of digit flexion could be <0% or >100% if subjects extended the digit past neutral or flexed it past the maximal passive flexion obtained during sensor calibration. For main outcomes, within-group descriptive data and between-group mean differences and inferential statistics are shown graphically (Fig 3A–3E) and reported in detail (Table 2). The amplitude of digit movement was not different between groups. Thumb and index finger cadences were lower in stroke than control subjects. Digit velocity was not different between groups. Thumb idle time was not different between groups, but index finger idle time was greater in stroke than control subjects. Likelihood ratio tests showed a significant effect of age on thumb cadence only; the 95% CI of the mean between-group difference for this outcome was adjusted for the effect of age. There were no effects of age on other outcomes.

**Table 2:** Within-group descriptive data and between-group differences of hand movement outcomes. Data are shown as mean (SD) unless otherwise stated. Cohen’s d (standardized mean difference) values are unitless.

	Thumb					Finger				
	Control	Stroke	Cohen’s d	Mean difference (95% CI)	P value	Control	Stroke	Cohen’s d	Mean difference (95% CI)	P value
Amplitude of movement (% flexion)	9 (3)	9 (4)	-0.06	0 (-3 to 2)	0.89	7 (2)	7 (2)	-0.19	0 (-2 to 1)	0.60
Cadence (movements/sec)	0.6 (0.3) [0.8]	0.4 (0.3) [0.2]	-1.12	-0.6 (-1.0 to -0.2)*^†^	0.003	0.9 (0.3)	0.4 (0.2)	-1.81	-0.5 (-0.7 to -0.3)*	<0.001
Velocity (% flexion/sec)	28 (13)	22 (11)	-0.50	-6 (-16 to 4)	0.21	24 (13)	16 (4)	-0.80	-7 (-14 to 0)	0.04
Percentage of idle time (%)	83 (5)	87 (8)	0.64	4 (-1 to 10)	0.11	78 (6)	84 (8)	0.88	6 (1 to 11)*	0.02
Longest idle time (sec)	204 (169)	576 (724)	0.72	373 (-37 to 782)	0.07	147 (92)	522 (647)	0.81	375 (29 to 721)*	0.04

Means adjusted by age are indicated in square brackets.

* 95% CI lie to one side of 0

^†^ 95% CI adjusted for significant effect of age (p = 0.045)

Cadences and velocities of the able-bodied subject’s thumb and finger during the typing task were calculated (Table 3) and exploratory outcomes of within-group descriptive data are reported (Table 4). In general, digit cadence was slow, and digit velocities were mostly between slow and medium speeds.

**Table 3:** Self-selected cadence and velocity of thumb and finger movements of an able-bodied subject while typing at slow, medium and fast speeds. Digit velocities correspond approximately to the following counts (cpm) or words per minute (wpm): slow (135 cpm, 25 wpm), medium (245 cpm, 50 wpm), fast (420 cpm, 85 wpm).

		Thumb	Finger
Cadence (movements/sec)	Slow	1.17	0.96
	Medium	1.62	2.42
	Fast	2.63	4.12
Velocity (% flexion/sec)	Slow	0.11	0.11
	Medium	0.36	0.2
	Fast	0.6	0.32

**Table 4:** Percentage of time that digits spend at a cadence or velocity range. Ranges were determined based on typing speeds of an able-bodied subject. Data are shown as mean (SD). Cohen’s d (standardized mean difference) values are unitless.

		Thumb			Finger		
		Control	Stroke	Cohen’s d	Control	Stroke	Cohen’s d
Percentage of time spent at a cadence range (%)	<slow	86 (15)	95 (6)	0.76	61 (18)	88 (15)	1.62
	≥ slow to <medium	8 (9)	4 (6)	-0.49	36 (16)	12 (15)	-1.53
	≥ medium to <fast	4 (6)	0 (1)	-0.94	3 (5)	0 (0)	-0.90
	≥ fast	1 (3)	0 (0)	-0.45	0 (0)	0 (0)	-0.37
Percentage of time spent at a velocity range (%)	<slow	48 (32)	70 (29)	0.71	19 (16)	48 (26)	1.33
	≥ slow to <medium	33 (19)	25 (26)	-0.39	41 (18)	34 (15)	-0.44
	≥ medium to <fast	16 (20)	5 (4)	-0.80	26 (20)	16 (14)	-0.61
	≥ fast	2 (4)	1 (2)	-0.38	14 (25)	2 (2)	-0.63

Post-hoc analyses in subjects with stroke showed that for some outcomes, digit movement differed between dominant and non-dominant hands (S1 Table) and those with greater hand function had better digit movement (S2 Table). Our study was not powered to detect effects in this post-hoc analysis; this is evident in the small sizes of effects and wide confidence intervals for many of the outcomes. For example, they correspond to fractions of 1% of digit flexion for the relevant outcomes. Consequently, these findings should be interpreted with caution.

## Discussion

We used stretch sensors to compare thumb and index finger movement over an extended period of time in people with stroke and able-bodied people. While the amplitudes of digit movement and digit velocities were not different between groups, people with stroke had lower thumb and finger cadences, and the finger was idle more often than the thumb. Exploratory findings show that in general, digit cadences and velocities ranged from slow to medium speeds.

Our findings should be interpreted in context of the following methodological considerations. First, digit movements were expressed as a percentage of maximal digit flexion, but maximal digit flexion depends on the sensor raw values obtained during calibration and whether the sensors retracted slightly during the trial. To obtain representative digit range of motion over the trial, we calculated maximal digit flexion range of motion using calibration files recorded at the start and end of the trial. Second, the exploratory findings may be less generalizable across subjects because digit movement cut-offs were determined relative to a single subject, and the typing task is unlikely to reflect motor performance across the full spectrum of functional hand tasks. Nevertheless, we report these findings to illustrate how digit movement recorded during our study relate to those produced during a reference functional task. Third, we could not capture movement of the distal interphalangeal joint of the index finger; pilot testing showed that the end of the stretch sensor could not be adequately secured to the end of the finger. Fourth, digit movement may have been influenced by the sensation of the sensors on the digits. A few subjects mentioned they would limit the movement of the tested hand at times because they could feel the sensors. However, since this bias is likely to have affected both groups of subjects, it would be accounted for in comparisons of outcomes between groups.

We found between-group differences for some characteristics of hand movement, but not others. On average, thumb and finger cadences were respectively 0.6 and 0.5 movements/sec lower in stroke than control subjects. Confidence intervals of both estimates are well under the line of no difference at 0, although the estimate was more precise for the finger. However, thumb and finger velocities were not different between groups. Cadence refers to the rate at which the digit moves (expressed in movements/sec) whereas velocity refers to the rate of change of digit movement (expressed in % flexion/second). Slower cadences in stroke subjects at comparable velocities mean that in general, stroke subjects covered a greater percentage of digit flexion for each movement compared to control subjects. That is, stroke subjects had longer digit “stride lengths” compared to controls. This finding seems counterintuitive, especially since the amplitudes of digit movement were not different between groups. The slower cadences may occur because the digits try to overcome mechanical coupling by adjacent digits [9, 33] or unwanted muscle co-contraction during movement [8].

On average, the finger was idle more often in stroke than control subjects (between-group mean difference: percentage of idle time 6%, longest idle time 375 seconds) but the thumb was not, and estimates for both outcomes of finger idle time were less precise. Idle time refers to time during which the digit was not moving. That is, the sensors detected no change in raw values of at least 1 unit. These findings are broadly consistent with findings from other studies showing that people with stroke use their affected arm much less compared to able-bodied people [18, 19, 26, 34]. However, it is difficult to determine whether neurophysiological changes or learned non-use contribute more to decreased use of the hand. Indeed, constraint-induced movement therapies designed to minimize learned non-use do not seem to improve hand function after stroke [17]. While finger idle time was greater in stroke than control subjects, it is surprising that on average, percentages of thumb and finger idle time were high in control subjects (>80% of total time). This suggests the hand does not need to move frequently to maintain normal hand dexterity and function, although stroke subjects may need more frequent hand movement to regain normal function. The thumb may not have been more idle in stroke than control subjects because it is involved in nearly all dexterous hand movements, whereas the index finger is not necessarily involved to the same extent and in the same manner. These findings suggest rehabilitation after stroke should encourage more frequent movement of the digits, with shorter periods of non-use.

This is the first study to quantify characteristics of thumb and finger movement over an extended period of time in people with stroke compared to able-bodied people. Although wearable sensors have been used in stroke rehabilitation to encourage overall upper limb movement [35], previous studies that specifically examined digit movement only reported flexion at a single digit joint [25]. This study adds new information by measuring digit movement using state-of-the-art technology, and quantifying different aspects of digit movement (amplitude, cadence, velocity, idle time) in a similar vein to studies investigating complex movements such as gait. Most large-sample stroke studies examined arm movement as far as the wrist. By quantifying movement at the digits distal to the wrist, this study shows how and to what extent people with stroke move the digits less often, over an extended period of time. That is, our study provides the first comprehensive, in-depth data on how much the hand is used after stroke. We deliberately recruited broadly to include stroke subjects with acute and sub-acute stroke who had a range of impaired hand function so our findings are generalizable to a broader range of people with stroke. We also measured the combined motion of different joints at the thumb and index finger to indicate movement of the whole digit.

Some limitations of this study are that we did not record whether the digits were moved actively or passively, nor the activities subjects performed while wearing the sensors, and some stroke subjects could not wear the sensors for the full 4 hours because they found them uncomfortable. While it would be useful to determine whether stroke subjects moved their hands actively or passively, accurately relating digit movement and muscle activity is complex because of weakness and involuntary muscle activity (e.g. spastic dystonia and co-contraction) common after stroke [36]. Nevertheless, the addition of electromyography (EMG) sensors together with sensors that detect movement would allow future studies to determine whether flexor and extensor muscles of the thumb and index finger are active or at rest during recorded movements, and identify the mechanisms of reduced cadence after stroke. The addition of video data of the activities subjects performed while wearing the sensors would also identify how digit movements correspond to specific activities. It would also be useful to know what proportion of movements took place in the context of formal rehabilitation. In patients with poor hand function where therapists aim to prevent muscle contractures, therapy tends to focus on slow, sustained stretches. These stretches would be reflected in our recordings as extremely low cadence, high amplitude movements. Depending on the amount of spastic dystonia present, involuntary muscle activity may or may not be present during these stretches. In patients with better hand function, therapy tends to focus on functional reaching and grasping tasks performed actively by the patient. Even so, we suspect the number of repetitions is relatively low and digit movement during these tasks would be slow. Thus, maintaining a simple activity log while measuring kinematic and EMG data could help identify whether increased hand movement occurs during rehabilitation compared to activities of daily living. Lastly, somatosensory deficits are associated with poor hand function [37], which may be associated with decreased and slower movements of the thumb and finger as we found. The association between spasticity and hand function is less clear [4, 38].

## Conclusions

Our findings add new information to the literature by providing the first comprehensive description of thumb and finger movement over an extended period of time in people with stroke. The clinical implications from this study are that rehabilitation after stroke should encourage the performance of functional tasks that involve movements at faster cadences, and encourage more frequent movement of the digits with shorter periods of inactivity. These strategies aim to improve and restore hand function after stroke.

## Supporting information

S1 Table Supplementary Table 1. Effect of testing the non-dominant hand compared to the dominant hand in subjects with stroke.
(PDF)

S2 Table Supplementary Table 2. Effect of hand motor function on outcomes in subjects with stroke.
(PDF)

## References

[1] Fergusson D, Hutton B, Drodge A. The epidemiology of major joint contractures: a systematic review of the literature. Clinical Orthopaedics and Related Research. 2007;456:22–29. 10.1097/BLO.0b013e3180308456 17179779

[2] Kwah LK, Harvey LA, Diong JH, Herbert RD. Half of the adults who present to hospital with stroke develop at least one contracture within six months: an observational study. Journal of Physiotherapy. 2012;58(1):41–47. 10.1016/S1836-9553(12)70071-1 22341381

[3] Kwah LK, Harvey LA, Diong J, Herbert RD. Models containing age and NIHSS predict recovery of ambulation and upper limb function six months after stroke: an observational study. Journal of Physiotherapy. 2013;59(3):189–197. 10.1016/S1836-9553(13)70183-8 23896334

[4] Kwah L, Herbert R. Prediction of walking and arm recovery after stroke: a critical review. Brain Sciences. 2016;6(4):E53 10.3390/brainsci6040053 27827835 PMC5187567

[5] Buma F, Kwakkel G, Ramsey N. Understanding upper limb recovery after stroke. Restorative Neurology and Neuroscience. 2013;31(6):707–22. 10.3233/RNN-130332 23963341

[6] Kreisel SH, Bazner H, Hennerici MG. Pathophysiology of stroke rehabilitation: temporal aspects of neuro-functional recovery. Cerebrovascular Diseases. 2006;21(1-2):6–17. 10.1159/000089588 16282685

[7] Kreisel SH, Hennerici MG, Bazner H. Pathophysiology of stroke rehabilitation: the natural course of clinical recovery, use-dependent plasticity and rehabilitative outcome. Cerebrovascular Diseases. 2007;23(4):243–255. 10.1159/000098323 17192704

[8] Triandafilou KM, Fischer HC, Towles JD, Kamper DG, Rymer WZ. Diminished capacity to modulate motor activation patterns according to task contributes to thumb deficits following stroke. Journal of Neurophysiology. 2011;106(4):1644–1651. 10.1152/jn.00936.2010 21753022

[9] Kamper DG, Fischer HC, Conrad MO, Towles JD, Rymer WZ, Triandafilou KM. Finger-thumb coupling contributes to exaggerated thumb flexion in stroke survivors. Journal of Neurophysiology. 2014;111(12):2665–2674. 10.1152/jn.00413.2013 24671534 PMC6442660

[10] McPherson JG, Chen A, Ellis MD, Yao J, Heckman CJ, Dewald JPA. Progressive recruitment of contralesional cortico-reticulospinal pathways drives motor impairment post stroke. Journal of Physiology. 2018;7:1211–1225. 10.1113/JP274968 PMC5878212 29457651

[11] Langhorne P, Bernhardt J, Kwakkel G. Stroke rehabilitation. Lancet. 2011;377(9778):1693–1702. 10.1016/S0140-6736(11)60325-5 21571152

[12] Serrada I, McDonnell MN, Hillier SL. What is current practice for upper limb rehabilitation in the acute hospital setting following stroke? A systematic review. NeuroRehabilitation. 2016;39(3):431–438. 10.3233/NRE-161374 27589513

[13] National Stroke Foundation. Clinical Guidelines for Stroke Management 2017. Melbourne, Australia; 2017.

[14] Taub E, Uswatte G, Mark VW, Morris DMM. The learned nonuse phenomenon: implications for rehabilitation. Europa Medicophysica. 2006;42(3):241–56. 17039223

[15] Wolf SL. Revisiting constraint-induced movement therapy: are we too smitten with the mitten? Is all nonuse “learned”? And other quandaries. Physical Therapy. 2007;87(9):1212–1223. 10.2522/ptj.20060355 17609329

[16] Kwakkel G, Veerbeek JM, van Wegen EEH, Wolf SL. Constraint-induced movement therapy after stroke. Lancet Neurology. 2015;14(2):224–234. 10.1016/S1474-4422(14)70160-7 25772900 PMC4361809

[17] Langhorne P, Coupar F, Pollock A. Motor recovery after stroke: a systematic review. Lancet Neurology. 2009;8(8):741–754. 10.1016/S1474-4422(09)70150-4 19608100

[18] Rand D, Eng JJ. Disparity between functional recovery and daily use of the upper and lower extremities during subacute stroke rehabilitation. Neurorehabilitation and Neural Repair. 2012;26(1):76–84. 10.1177/1545968311408918 21693771 PMC3233607

[19] Strommen AM, Christensen T, Jensen K. Quantitative measurement of physical activity in acute ischemic stroke and transient ischemic attack. Stroke. 2014;45(12):3649–3655. 10.1161/STROKEAHA.114.006496 25370584

[20] Skarin M, Sjöholm A, Nilsson ÅL, Nilsson M, Bernhardt J, Lindén T. A mapping study on physical activity in stroke rehabilitation: establishing the baseline. Journal of Rehabilitation Medicine. 2013;45(10):997–1003. 10.2340/16501977-1214 24150662

[21] Lang CE, MacDonald JR, Reisman DS, Boyd L, Jacobson Kimberley T, Schindler-Ivens SM, et al Observation of amounts of movement practice provided during stroke rehabilitation. Archives of Physical Medicine and Rehabilitation. 2009;90(10):1692–1698. 10.1016/j.apmr.2009.04.005 19801058 PMC3008558

[22] Fini NA, Holland AE, Keating J, Simek J, Bernhardt J. How is physical activity monitored in people following stroke? Disability and Rehabilitation. 2015;37(19):1717–1731. 10.3109/09638288.2014.978508 25374044

[23] Gentner R, Classen J. Development and evaluation of a low-cost sensor glove for assessment of human finger movements in neurophysiological settings. Journal of Neuroscience Methods. 2009;178(1):138–147. 10.1016/j.jneumeth.2008.11.005 19056422

[24] Patel S, Park H, Bonato P, Chan L, Rodgers M. A review of wearable sensors and systems with application in rehabilitation. Journal of Neuroengineering and Rehabilitation. 2012;9:21 10.1186/1743-0003-9-21 22520559 PMC3354997

[25] Simone LK, Sundarrajan N, Elovic EP, Luo X, Jia Y, Kamper DG. Measuring finger flexion and activity trends over a 25 hour period using a low cost wireless device. Conference Proceedings IEEE Engineering in Medicine and Biology Society. 2006;1:6281–6284. 10.1109/IEMBS.2006.260546 17945949

[26] Rowe JB, Friedman N, Chan V, Cramer SC, Bachman M, Reinkensmeyer DJ. The variable relationship between arm and hand use: a rationale for using finger magnetometry to complement wrist accelerometry when measuring daily use of the upper extremity. Conference Proceedings IEEE Engineering in Medicine and Biology Society. 2014;2014:4087–4090. 10.1109/EMBC.2014.6944522 25570890

[27] Liu X, Rajan S, Ramasarma N, Bonato P, Lee SI. Finger-worn sensors for accurate functional assessment of the upper limbs in real-world settings. In: Conference Proceedings IEEE Engineering in Medicine and Biology Society. IEEE; 2018. p. 4440–4443. 10.1109/EMBC.2018.8513134 30441336

[28] Liu X, Rajan S, Ramasarma N, Bonato P, Lee SI. The use of a finger-worn accelerometer for monitoring of hand use in ambulatory settings. IEEE Journal of Biomedical and Health Informatics. 2018; p. 1–1. 10.1109/JBHI.2018.2821136 29994103

[29] Furudate Y, Yamamoto K, Chiba K, Ishida Y, Mikami S. Quantification method of motor function recovery of fingers by using the device for home rehabilitation. Conference Proceedings IEEE Engineering in Medicine and Biology Society. 2017;2017:3872–3875. 10.1109/EMBC.2017.8037702 29060743

[30] Kwakkel G, Lannin NA, Borschmann K, English C, Ali M, Churilov L, et al Standardized measurement of sensorimotor recovery in stroke trials: Consensus-based core recommendations from the Stroke Recovery and Rehabilitation Roundtable. International Journal of Stroke. 2017;12(5):451–461. 10.1177/1747493017711813 28697709

[31] Carr JH, Shepherd RB, Nordholm L, Lynne D. Investigation of a new motor assessment scale for stroke patients. Physical Therapy. 1985;65(2):175–180. 10.1093/ptj/65.2.175 3969398

[32] Lannin NA. Reliability, validity and factor structure of the upper limb subscale of the Motor Assessment Scale (UL-MAS) in adults following stroke. Disability and Rehabilitation. 2004;26(2):109–116. 10.1080/0963828032000157970 14668148

[33] Li S, Latash ML, Yue GH, Siemionow V, Sahgal V. The effects of stroke and age on finger interaction in multi-finger force production tasks. Clinical Neurophysiology. 2003;114(9):1646–55. 10.1016/S1388-2457(03)00164-0 12948793

[34] Kaur G, English C, Hillier S. How physically active are people with stroke in physiotherapy sessions aimed at improving motor function? A systematic review. Stroke Research and Treatment. 2012;2012:820673 10.1155/2012/820673 22567542 PMC3337516

[35] Lee SI, Adans-Dester CP, Grimaldi M, Dowling AV, Horak PC, Black-Schaffer RM, Bonato P, Gwin JT Enabling stroke rehabilitation in home and community settings: a wearable sensor-based approach for upper-limb motor training. IEEE Journal of Translational Engineering in Health and Medicine. 2018 10.1109/JTEHM.2018.2829208 PMC5951609 29795772

[36] Baude M, Nielsen JB, Gracies JM. The neurophysiology of deforming spastic paresis: A revised taxonomy. Annals of Physical and Rehabilitation Medicine. 2018 10.1016/j.rehab.2018.10.004 30500361

[37] Meyer S, Karttunen AH, Thijs V, Feys H, Verheyden G. How do somatosensory deficits in the arm and hand relate to upper limb impairment, activity, and participation problems after stroke? A systematic review. Physical Therapy. 2014;94(9):1220–1231. 10.2522/ptj.20130271 24764072

[38] Allison R, Kilbride C, Chynoweth J, Creanor S, Frampton I, Marsden J. What is the longitudinal profile of impairments and can we predict difficulty caring for the profoundly affected arm in the first year poststroke? Archives of Physical Medicine and Rehabilitation. 2018;99(3):433–442. 10.1016/j.apmr.2017.07.016 28866012

