# Supplementary material for: Thumb and finger movement is reduced after stroke: An observational study
Source: PLoS One. 2019 Jun 12;14(6):e0217969. doi: 10.1371/journal.pone.0217969 (PMC6561636; doi:10.1371/journal.pone.0217969)
Supplement: S1 Table — Effect of testing the non-dominant hand compared to the dominant hand in subjects with stroke. (PDF) [file pone.0217969.s001.pdf]

Supplementary Table 1. **Effect of testing the non-dominant hand compared to the dominant hand in subjects with stroke.** Data are shown as mean (SD) unless otherwise stated.

	Thumb				Finger			
	**Dominant (n=6)**	**Non-dominant (n=7)**	**Mean difference (95% CI)**	**p value**	**Dominant (n=7)**	**Non-dominant (n=8)**	**Mean difference (95% CI)**	**p value**
Amplitude of movement (% flexion)	6 (2)	11 (4)	5 (1 to 9)*	0.02	6 (2)	7 (2)	1 (-1 to 3)	0.30
Cadence (movements/sec)	0.3 (0.3)	0.4 (0.2)	0.2 (-0.2 to 0.5)	0.30	0.4 (0.2)	0.4 (0.3)	0 (-0.2 to 0.3)	0.80
Velocity (% flexion/sec)	16 (4)	28 (13)	12 (1 to 24)*	0.04	15 (3)	17 (4)	2 (-2 to 6)	0.36
Percentage of idle time (%)	90 (7)	84 (7)	-7 (-16 to 2)	0.14	85 (7)	83 (9)	-2 (-11 to 6)	0.58
Longest idle time (sec)	950 (897)	256 (342)	-695 (-1497 to 108)	0.08	770 (846)	305 (329)	-465 (-1162 to 232)	0.17

Data are shown as mean (SD) unless otherwise stated.
* 95% CI lie to one side of 0
