# Supplementary material for: Thumb and finger movement is reduced after stroke: An observational study
Source: PLoS One. 2019 Jun 12;14(6):e0217969. doi: 10.1371/journal.pone.0217969 (PMC6561636; doi:10.1371/journal.pone.0217969)
Supplement: S2 Table — Effect of hand motor function on outcomes in subjects with stroke. (PDF) [file pone.0217969.s002.pdf]

Supplementary Table 2. **Effect of hand motor function on outcomes in subjects with stroke.** Hand motor function is measured using the Motor Assessment Scale (MAS). Data are shown as mean (SD) unless otherwise stated.

	**Thumb (n=13)**				**Finger (n=15)**			
	**MAS 7 mean effect (95% CI)**	**P value**	**MAS 8 mean effect (95% CI)**	**P value**	**MAS 7 mean effect (95% CI)**	**P value**	**MAS 8 mean effect (95% CI)**	**P value**
Amplitude of movement (% flexion)	0.1 (-0.8 to 1.0)	0.86	0.2 (-0.8 to 1.2)	0.66	0.5 (0.3 to 0.8)*	0.001	0.5 (0.2 to 0.8)*	0.005
Cadence (movements/sec)	0.08 (0.05 to 0.11)*	<0.001	0.09 (0.06 to 0.11)*	<0.001	0.06 (0.02 to 0.10)*	0.004	0.07 (0.03 to 0.11)*	0.002
Velocity (% flexion/sec)	0.2 (-2.4 to 2.7)	0.88	1.0 (-2.1 to 3.4)	0.60	0.9 (0.4 to 1.5)*	0.003	1.0 (0.5 to 1.6)*	0.002
Percentage of idle time (%)	-2.0 (-3.2 to -0.9)*	0.003	-2.1 (-3.5 to -0.8)*	0.005	-1.6 (-2.9 to -0.3)*	0.021	-1.8 (-3.2 to -0.3)*	0.019
Longest idle time (sec)	-165 (-288 to -42)*	0.013	-146 (-296 to 4)	0.055	-123 (-240 to -6)*	0.04	-115 (-249 to 19)	0.086

* 95% CI lie to one side of 0
